# Supplementary material for: Personal protective equipment for reducing the risk of COVID-19 infection among healthcare workers involved in emergency trauma surgery during the pandemic: an umbrella review protocol
Source: BMJ Open. 2021 Mar 2;11(3):e045598. doi: 10.1136/bmjopen-2020-045598 (PMC7929636; doi:10.1136/bmjopen-2020-045598)
Supplement: Supplementary data [file bmjopen-2020-045598supp001.pdf]

Appendix I: The Living Overview of Evidence (L·OVE) search for COVID-19 studies involving N95 masks in healthcare workers

LoVE COVID-19 platform: https://app.iloveevidence.com/loves/5e6fdb9669c00e4ac072701d

Prevention or Treatment → Procedures → Protective measures → Personal Protective Equipment → (Options: Gloves, Shoe Covers, Masks, Eye Protection, Head Coverings, Intubation box) → Masks → Facemasks → N95 Masks

Population → Health workers → (can choose specific healthcare settings)

Results: 67 total articles included
- Broad syntheses: 3
- Systematic reviews: 10
- Primary studies: 54

The methods and report of the Special L·OVE of COVID-19 can be found using the following link: https://app.iloveevidence.com/loves/5e6fdb9669c00e4ac072701d?question_domain=5b1dcd8ae611de7ae84e8f14&population=5e9cae0f69c00e502f84a41c&intervention=5edcde1269c00e3d6aae4587&section=methods&classification=all
