# Supplementary material for: Personal protective equipment for reducing the risk of COVID-19 infection among healthcare workers involved in emergency trauma surgery during the pandemic: an umbrella review protocol
Source: BMJ Open. 2021 Mar 2;11(3):e045598. doi: 10.1136/bmjopen-2020-045598 (PMC7929636; doi:10.1136/bmjopen-2020-045598)
Supplement: Author's manuscript [file bmjopen-2020-045598.draft_revisions.pdf]

BMJ Open is committed to open peer review. As part of this commitment we make the peer review history of every article we publish publicly available.

When an article is published we post the peer reviewers' comments and the authors' responses online. We also post the versions of the paper that were used during peer review. These are the versions that the peer review comments apply to.

The versions of the paper that follow are the versions that were submitted during the peer review process. They are not the versions of record or the final published versions. They should not be cited or distributed as the published version of this manuscript.

BMJ Open is an open access journal and the full, final, typeset and author-corrected version of record of the manuscript is available on our site with no access controls, subscription charges or pay-per-view fees (http://bmjopen.bmj.com).

If you have any questions on BMJ Open's open peer review process please email info.bmjopen@bmj.com

BMJ Open

Personal protective equipment for reducing the risk of COVID-19 infection among healthcare workers involved in emergency trauma surgery during the pandemic: a systematic review protocol

Journal:	*BMJ Open*
Manuscript ID	bmjopen-2020-045598
Article Type:	Protocol
Date Submitted by the Author:	06-Oct-2020
Complete List of Authors:	Griswold, Dylan; Cambridge University, Clinical Neurosciences<br>Gempeler, Andres; Fundación Valle del Lili, Clinical Research<br>Kolias, Angelos; Cambridge University, Neurosciences and Neurosurgery<br>Hutchinson, Peter; Cambridge University, Neurosciences and Neurosurgery<br>Rubiano, Andres M; Universidad El Bosque, Neurosciences Institute
Keywords:	COVID-19, NEUROSURGERY, ORTHOPAEDIC & TRAUMA SURGERY, Trauma management < ORTHOPAEDIC & TRAUMA SURGERY, SURGERY

SCHOLARONE™
Manuscripts

I, the Submitting Author has the right to grant and does grant on behalf of all authors of the Work (as defined in the below author licence), an exclusive licence and/or a non-exclusive licence for contributions from authors who are: i) UK Crown employees; ii) where BMJ has agreed a CC-BY licence shall apply, and/or iii) in accordance with the terms applicable for US Federal Government officers or employees acting as part of their official duties; on a worldwide, perpetual, irrevocable, royalty-free basis to BMJ Publishing Group Ltd ("BMJ") its licensees and where the relevant Journal is co-owned by BMJ to the co-owners of the Journal, to publish the Work in this journal and any other BMJ products and to exploit all rights, as set out in our licence.

The Submitting Author accepts and understands that any supply made under these terms is made by BMJ to the Submitting Author unless you are acting as an employee on behalf of your employer or a postgraduate student of an affiliated institution which is paying any applicable article publishing charge ("APC") for Open Access articles. Where the Submitting Author wishes to make the Work available on an Open Access basis (and intends to pay the relevant APC), the terms of reuse of such Open Access shall be governed by a Creative Commons licence – details of these licences and which Creative Commons licence will apply to this Work are set out in our licence referred to above.

Other than as permitted in any relevant BMJ Author's Self Archiving Policies, I confirm this Work has not been accepted for publication elsewhere, is not being considered for publication elsewhere and does not duplicate material already published. I confirm all authors consent to publication of this Work and authorise the granting of this licence.

For peer review only

1

Personal protective equipment for reducing the risk of COVID-19 infection among healthcare workers involved in emergency trauma surgery during the pandemic: a systematic review protocol

Dylan P Griswold, BA[1,2]dg550@cam.ac.uk **Andres Gempeler, MD**[3]andres.gempeler@fvl.org.co
Angelos Kolias, MD, PhD[1,2]ak721@cam.ac.uk **Peter J. Hutchinson, MBBS, PhD, FRCS**[1,2]pjah2@cam.ac.uk
Andres M. Rubiano, MD, PhD[1,4,5]
1 NIHR Global Health Research Group on Neurotrauma, University of Cambridge, Cambridge, UK
2 Division of Neurosurgery, Department of Clinical Neurosciences, Addenbrooke's Hospital & University of Cambridge, Cambridge, UK 3 Clinical Research Center, Fundación Valle del Lili, Cali, Colombia4 Neuroscience Institute, INUB-MEDITECH Research Group, El Bosque University, Bogotá, Colombia 5 Neurological Surgery Service, Vallesalud Clinic, Cali, Colombia.

Keywords:systematic review; broad-evidence review; COVID-19; global health; trauma surgery; evidence-based practice; PPE; N95 masks.

Word count: 1875

Correspondence:
Andres M. Rubiano
Neuroscience Institute, INUB-MEDITECH Research Group
El Bosque University
Bogotá, Colombia
andresrubiano@aol.com

For peer review only

2

ABSTRACT

Introduction: Many healthcare facilities in low-and middle-income countries are inadequately resourced and may lack optimal organization and governance, especially concerning surgical health systems. COVID-19 has the potential to decimate these already strained surgical healthcare services unless health systems take stringent measures to protect healthcare workers from viral exposure and ensure the continuity of specialized care for the patients. The objective of this broad evidence synthesis is to identify and summarize the available literature regarding the efficacy of different personal protective equipment (PPE) for reducing the risk of COVID-19 infection in health personnel caring for patients undergoing trauma surgery in low-resource environments (LREs).

MethodsWe will conduct several searches in the L·OVE (Living OVerview of Evidence) platform for COVID-19, a system that performs automated regular searches in PubMed, Embase, Cochrane Central Register of Controlled Trials (CENTRAL), and over thirty other sources. The search results will be presented according to the Preferred Reporting Items for Systematic Reviews and Meta-Analyses (PRISMA) flow diagram. This review will preferentially consider systematic reviews of experimental and quasi-experimental studies, as well as individual studies of such designs, evaluating the effect of different PPE on the risk of COVID-19 infection in healthcare workers (HCWs) involved in emergency trauma surgery. Critical appraisal of the eligible studies for methodological quality will be conducted. Data will be extracted using the standardized data extraction tool in Covidence. Studies will, when possible, be pooled in a statistical meta-analysis using JBI SUMARI. The Grading of Recommendations, Assessment, Development, and Evaluation (GRADE) approach for grading the certainty of evidence will be followed, and a Summary of Findings (SoF) will be created.

Ethics and dissemination Ethical approval is not required for this review. The plan for dissemination is to publish review findings in a peer-reviewed journal and present findings at high-level conferences that engage the most pertinent stakeholders.

PROSPERO registration numberCRD42020198267

For peer review only

3

ARTICLE SUMMARY

Strengths and Limitations of this study

- To the best of our knowledge this protocol provides a detailed description of the first systematic review on the effects of PPE for protection of emergency trauma surgery staff against COVID-19 infection
- The protocol adheres to the Preferred Reporting Items for Systematic Reviews and Meta-Analyses Protocols guidelines for reporting a systematic review protocol
- The protocol is being conducted by a multidisciplinary team with extensive experience of conducting high-quality systematic reviews
- Given the rate at which new COVID-19 related studies are being published, there is the possibility that new studies will have been published at the time of review publication that were not available at the time of writing the review

For peer review only

4

-

INTRODUCTION

Many healthcare facilities in low-and middle-income countries are inadequately resourced. COVID-19 has the potential to decimate these already strained surgical healthcare services unless health systems take stringent measures to protect healthcare workers (HCWs) from viral exposure. A recent study showed that 15.6% of confirmed COVID-19 patients are symptomatic and that nearly half of patients with no symptoms at detection time will develop symptoms later.[1] Furthermore, the preoperative evaluation of emergency trauma patients is limited. These factors impede and confound diagnostic triage. Improper infection prevention may create a 'super-spreader' event in a high-volume healthcare facility or reduce available personnel. Consequently, the infection control strategy of trauma surgery staff is a top priority.

To take care of patients, providers must first take care of themselves. Personal protective equipment (PPE) is paramount to protect health care workers from contracting the virus and becoming disease carriers. Basic recommended PPE for trauma surgery staff of high-income country facilities include: 1) a surgical mask or better for all personnel interacting with patients and in the OR (including cleaning staff); 2) N95 or better mask for all staff in close contact with the patients (<6 feet away); 3) PAPR for aerosolising and high-risk procedures (ear, nose, throat, thoracic, and transsphenoidal neurosurgery operations); 4) universal testing of patients pre-operatively to enable appropriate PPE use, and 5) changing scrubs after every procedure.[2] These recommendations are suitable for high-resource settings but are less feasible in low-resource settings. A rapid-turnaround survey of 40 healthcare organisations across 15 LMICs revealed that 70% lack PPE and COVID-19 testing kits, and only 65% of the respondents showed confidence in hospital staff's knowledge about precautions to be taken to prevent COVID-19 infection among hospital personnel.[3]

Some resource-adjusted recommendations include the use of cloth masks and bandanas. While innovative, their moisture retention, reusability, and filtration are considered very inferior to N95, and other masks.[4] What is most needed is evidence guided recommendations for PPE use and COVID-19 screening in LMICs surgical systems where resources are either limited or unavailable. HCWs have been instructed to consider refraining from caring for patients in the absence of adequate PPE availability.

A preliminary search of PROSPERO, MEDLINE, the Cochrane Database of Systematic Reviews, and the JBI Database of Systematic Reviews and Implementation Reports was conducted, and no current or underway systematic reviews on the topic were identified.

The primary objective of the review is to summarise the effects of different personal protective equipment in reducing the risk of COVID-19 infection of health personnel caring for patients undergoing trauma surgery. The purpose of the review is to inform recommendations for the rational use of PPE in emergency surgery staff, particularly in low resources environments where PPE shortages and high costs are expected to hamper the safety of HCWs and affect the care of trauma patients. A preliminary search of PROSPERO, MEDLINE, the Cochrane Database of Systematic Reviews, and the JBI Database of Systematic Reviews and Implementation Reports was conducted, and no current or underway systematic reviews on the topic were identified.

METHODS

Protocol registration

A protocol of this review following the PRISMA statement was registered in the International Prospective Registry of Systematic Reviews (PROSPERO; CRD42020198267). Any changes to the protocol will be amended in PROSPERO and reported in the final review. This review was conducted following the JBI methodology for systematic reviews.[5] The protocol adheres to the Preferred Reporting Items for Systematic Reviews and Meta-Analyses Protocols (PRISMA-P) 2015.[6]

For peer review only

Patient and public involvement

Patients and the public were not involved in the design of this systemic review protocol.

Study design

A systematic review of peer review and grey literature following the Preferred Reporting Items for Systematic Reviews and Meta-Analyses (PRISMA) approach by Moher *et al*[7]is planned for this review. Figure 1 summarises the planned stages of the review as described in this protocol.

Data source and search strategy

We will conduct several searches in the L·OVE (Living OVerview of Evidence) platform for COVID-19, a system that performs automated regular searches in PubMed, Embase, Cochrane Central Register of Controlled Trials (CENTRAL), and over thirty other sources. When compared to manual searches, this platform consistently identifies all the available studies associated with the terms of interest. It allows for a fast (automated) search that is easy to update - a crucial element given the urgent need to answer the research question rapidly and thoroughly. We will search for systematic reviews and randomized trials evaluating the effect of different personal protective equipment (PPE) on the risk of COVID-19 infection in personnel involved in emergency trauma surgery during the pandemic. Other in-hospital clinical settings will be considered for inclusion and synthesis if evidence for trauma surgery setting is not available. Different clinical settings will be treated as subgroups from which extrapolation will be possible when considered adequate. Non-randomized studies will be considered if systematic reviews and RCTs are not available or are scarce and of low quality. We will include preprint studies identified in our searches, but no ongoing studies will be considered. Ongoing studies will be counted as excluded studies in the corresponding tables and PRISMA diagram.

Selection of studies

Following the search, all identified citations will be collated and uploaded into EndNoteX9 (Clarivate Analytics, PA, USA). The citations will then be imported into JBI SUMARI for the review process. Two independent reviewers will examine titles and abstracts for eligibility. The full text of selected studies will be retrieved and assessed. Full-text studies that do not meet the inclusion criteria will be excluded, and a list of such excluded studies will be provided. Disagreements between the reviewers during title and abstract screening or full-text screening, will be resolved by consensus, or with a third reviewer. The results of the search will be reported in full in the final report and presented in a Preferred Reporting Items for Systematic Reviews and Meta-analyses (PRISMA) flow diagram.[7]

Eligibility criteria

Inclusion criteria

Participants

The review will preferentially include studies involving healthcare workers in emergency trauma surgery during the COVID-19 pandemic. Given the likelihood that reports on this specific population are scarce or even non-existent, if not available or insufficient we will consider studies of healthcare workers in any procedural and in-hospital setting such as ER and critical care management in COVID-19. Studies summarizing the available evidence for other viral respiratory illnesses will be considered if COVID-19 evidence is not available and the setting reported is trauma surgery.

For peer review only

Intervention(s)

Different types of PPE used for treating patients requiring emergency trauma surgery.

Comparator(s)

Comparators of interest are no PPE use and different types of PPE.

Outcomes

This review will consider studies that include the following outcomes: Risk of contagion to health personnel involved in the care of the described population during the COVID-19 pandemic; differences in surgical field vision; expressed as incidence differences, risk ratios or odds ratios. All outcomes will be summarized narratively

Types of studies

This review will consider systematic reviews of experimental and observational studies, and experimental or observational studies if not included in systematic reviews that fulfilled population and intervention criteria. We will also include reports on implementation strategies that could inform recommendations for variable resource settings. Only studies published in English or Spanish will be included. We will include preprint studies identified in our search, but no ongoing studies will be considered.

Quality assessment of included studies

Eligible studies will be critically appraised by one reviewer and verified with the other. We will use the AMSTAR tool to assess the risk of bias in systematic reviews, the Cochrane risk of bias tool (RoB2) for RCTs, and the ROBINS-I tool for non-randomized studies.[8–10] Risk of Bias will be assessed only for the primary outcome: infection of healthcare workers. The results of the risk of bias assessment will be reported narratively and inform grading of evidence summarized in SoF tables. Disagreements will be solved by consensus or by a third reviewer.

Data extraction

Data will be extracted from the included studies by a reviewer and verified by a second reviewer using a data extraction tool from JBI SUMARI.[5] The data extracted will include specific details about the populations, study methods, interventions, and outcomes of significance to the review question and specific objectives. Disagreements will be solved by consensus.

Data synthesis

Studies will be summarized narratively. Effect sizes from systematic reviews and from individual studies not included in them, will be expressed as odds ratios (for dichotomous data) with their 95% confidence intervals. Decision rules regarding data extraction for situations where data are:

- reported at multiple time points: include all and note time of measurement for each.

- multiple “doses”: N/A – (all types of PPE will be recorded)

For peer review only

· multiple exposures are compared (e.g., ever exposed, frequency of exposure): as mentioned before, exposure will relate to the clinical setting of healthcare workers studied and will be classified accordingly.

For summarizing results from other settings different to trauma surgery, effect of PPE will be summarized by subgroups according to different clinical settings.

Assessing certainty in the findings

The Grading of Recommendations, Assessment, Development, and Evaluation (GRADE) approach for grading the certainty of evidence will be followed , and a Summary of Findings (SoF) will be created using online software GRADEPro GDT 2020 (McMaster University, ON, Canada).[11,12] The SoF will present the following information where appropriate: absolute risks for the treatment and control, estimates of relative risk, and quality of the evidence based on the risk of bias, directness, heterogeneity, precision, and risk of publication bias. The outcomes reported in the SoF will be risk of COVID-19 infection.

Ethics and dissemination

No ethical approval will be required, as this review is based on already published data and does not involve interaction with human subjects. The plan for dissemination, however, is to publish the findings of the review in a peer-reviewed journal and present findings at high- level international conferences that engage the most pertinent stakeholders. The proposed systematic review will provide a detailed summary of available evidence on the effects of different personal protective equipment in reducing the risk of COVID-19 infection of health personnel caring for patients undergoing trauma surgery. The purpose of the review is to inform recommendations for the rational use of PPE in emergency surgery staff, particularly in low resources environments where PPE shortages and high costs are expected to hamper the safety of HCWs and affect the care of trauma patients.

DISCUSSION

This protocol has been rigorously developed and designed specifically to assess the effects of different personal protective equipment in reducing the risk of COVID-19 infection of health personnel caring for patients undergoing trauma surgery. Given the limited recent evidence associated with the primary objective, findings from the review will be critical for researchers, policymakers, government and non-governmental organisations for planning and developing guideline recommendations for PPE use in emergency trauma surgery settings, especially in low-and middle-income countries. If protocol modifications are required, the authors will include the detailed description of any changes along with a justification during the publication of the review.

Clearly, in the era of the COVID-19, where protecting healthcare workers from infection is essential, up-to-date information on the effects of PPE for protection against COVID-19 infection is essential. This review will serve an important role as a repository of available evidence for the purpose of setting effective policy and clinical guideline recommendations.

For peer review only

8

Contributors AR, PJH, and AK conceived the review. DG, and AG designed the review. DG refined the review design. DG and AG were involved in the initial drafting of the manuscript. All authors were involved in subsequent draft manuscript reviews and updates and approved the final version of this protocol.

Funding This research was commissioned by the National Institute for Health Research (NIHR) Global Health Research Group on Neurotrauma (project 16/137/105) using UK aid from the UK Government. The views expressed in this publication are those of the author(s) and not necessarily those of the NIHR or the Department of Health and Social Care. Dr Hutchinson is the Chief Investigator of the RESCUEicp and RESCUE- ASDH randomised trials. Mr Griswold was supported by the Gates Cambridge Trust. Prof Hutchinson was supported by a Research Professorship from the NIHR, the NIHR Cambridge Biomedical Research Centre, a European Union Seventh Framework Programme grant (CENTER-TBI; grant no. 602,150), and the Royal College of Surgeons of England. Dr Kolias was supported by a Clinical Lectureship from the School of Clinical Medicine, University of Cambridge, and the Royal College of Surgeons of England. Dr Gempeler was supported by a grant from the Clinical Research Center, Fundación Valle del Lili.

Competing interests None declared.
Patient consent for publication Not required.

Data statement: This review is based on previously published data. Any relevant data will be published with the review as either an appendix or as an online supplement.

Acknowledgements None to report
Open access This is an open access article distributed in accordance with the Creative Commons Attribution Non Commercial (CC BY-NC 4.0) license, which permits others to distribute, remix, adapt, build upon this work non-commercially, and license their derivative works on different terms, provided the original work is properly cited, appropriate credit is given, any changes made indicated, and the use is non-commercial. See: http://creativecommons.org/licenses/by-nc/4.0/.

License to BMJ Group for Publication: I *Andres M. Rubiano*The Corresponding Author of this article contained within the original manuscript which includes any diagrams & photographs within and any related or stand-alone film submitted (the Contribution") has the right to grant on behalf of all authors and does grant on behalf of all authors, a licence to the BMJ Publishing Group Ltd and its licencees, to permit this Contribution (if accepted) to be published in the BMJ and any other BMJ Group products and to exploit all subsidiary rights, as set out in our licence set out at: http://www.bmj.com/about-bmj/resources-authors/forms-policies-and-checklists/copyright-open-access-and-permission-reuse.

For peer review only

9

REFERENCES

1 Zhu J, Ji P, Pang J, et al. Clinical characteristics of 3062 COVID-19 patients: A meta-analysis. J Med Virol2020;92:1902–14. doi:10.1002/jmv.25884

2 Stewart CL, Thornblade LW, Diamond DJ, et al. Personal Protective Equipment and COVID-19: A Review for Surgeons. Ann Surg 2020;272:e132–8. doi:10.1097/sla.0000000000003991

3 Gonzalez N, Hatchcock A, Jansen N, et al. A Rapid Turnaround Survey of COVID-19 in LMICs: The Readiness, Responses, and Challenges of Healthcare Organizations. 2020.

4 R C, T D, R J, et al. Masks for Prevention of Respiratory Virus Infections, Including SARS-CoV-2, in Health Care and Community Settings: A Living Rapid Review. Ann Intern Med Published Online First: 1 January 2020. doi:10.7326/m20-3213

5 Aromataris E, Munn Z, Tufanaru C, et al. JBI Reviewer's Manual. Published Online First: 2019. doi:10.46658/jbirm-17-03

6 Group P-P, Moher D, Shamseer L, et al. Preferred reporting items for systematic review and meta-analysis protocols (PRISMA-P) 2015 statement. Syst Rev 2015;4:1. doi:10.1186/2046-4053-4-1

7 Moher D, Liberati A, Tetzlaff J, et al. Preferred reporting items for systematic reviews and meta-analyses: the PRISMA statement. Int J Surg LondEngl2010;8:336–41. doi:10.1016/j.ijsu.2010.02.007

8 Sterne JAC, Savović J, Page MJ, et al. RoB 2: a revised tool for assessing risk of bias in randomised trials. Bmj 2019;366:l4898. doi:10.1136/bmj.l4898

9 Shea BJ, Reeves BC, Wells G, et al. AMSTAR 2: a critical appraisal tool for systematic reviews that include randomised or non-randomised studies of healthcare interventions, or both. Bmj Clin Res Ed 2017;358:j4008. doi:10.1136/bmj.j4008

10 Sterne JA, Hernán MA, Reeves BC, et al. ROBINS-I: a tool for assessing risk of bias in non-randomised studies of interventions. Bmj Clin Res Ed 2016;355:i4919. doi:10.1136/bmj.i4919

11 Atkins D, Best D, Briss PA, et al. Grading quality of evidence and strength of recommendations. Bmj2004;328:1490. doi:10.1136/bmj.328.7454.1490

12 Guyatt G, Oxman AD, Akl EA, et al. GRADE guidelines: 1. Introduction—GRADE evidence profiles and summary of findings tables. J Clin Epidemiol 2011;64:383–94. doi:10.1016/j.jclinepi.2010.04.026

For peer review only

10

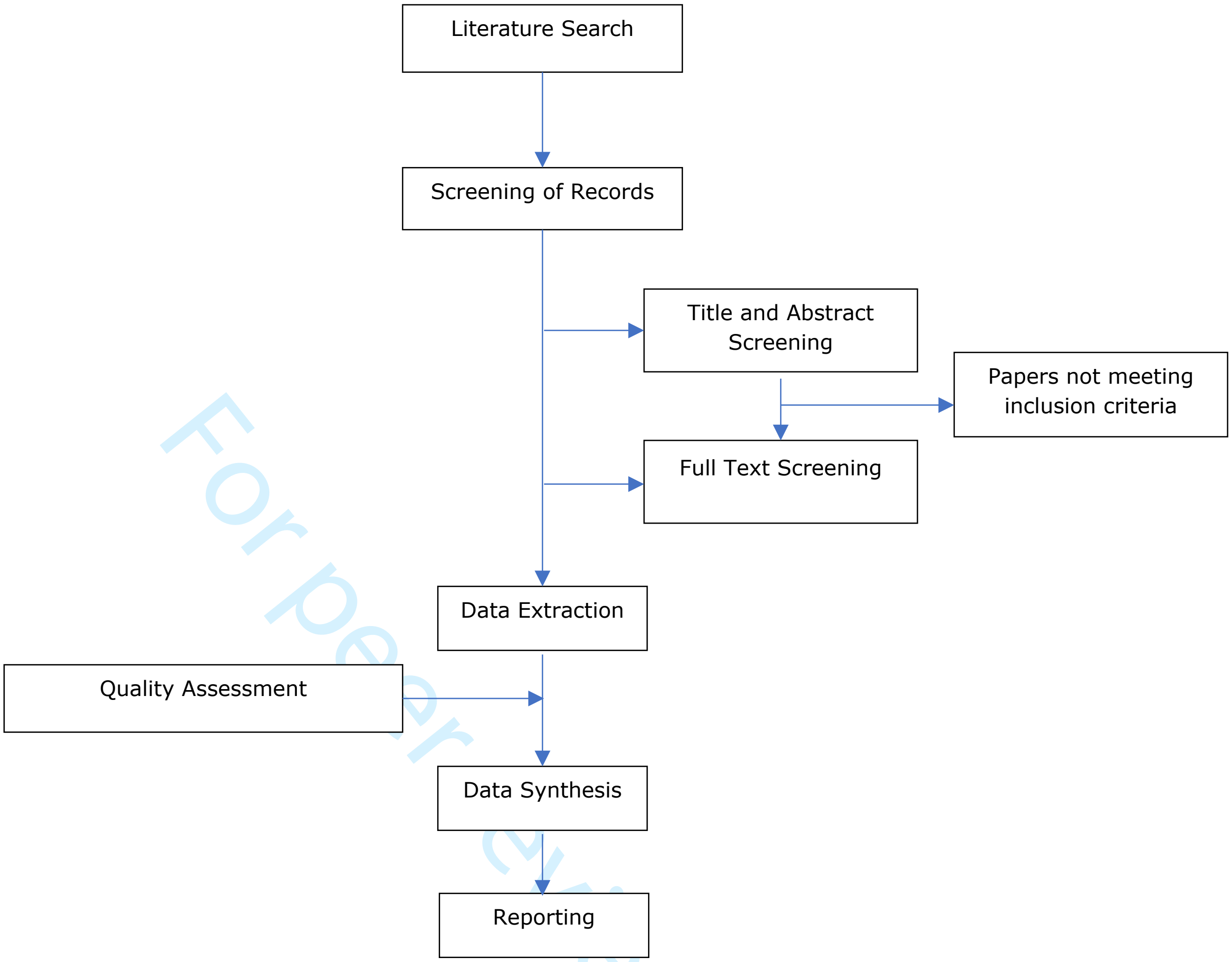

Figure 1 Summary of search strategy search process.

For peer review only

Reporting checklist for protocol of a systematic review.

		Reporting Item	Page Number
**Title**			
Identification	#1a	Identify the report as a protocol of a systematic review	1
Update	#1b	If the protocol is for an update of a previous systematic review, identify as such	NA
**Registration**			
	#2	If registered, provide the name of the registry (such as PROSPERO) and registration number	1
**Authors**			
Contact	#3a	Provide name, institutional affiliation, e-mail address of all protocol authors; provide physical mailing address of corresponding author	1
Contribution	#3b	Describe contributions of protocol authors and identify the guarantor of the review	7
**Amendments**			
	#4	If the protocol represents an amendment of a previously completed or published protocol, identify as such and list	6

For peer review only

		changes; otherwise, state plan for documenting important protocol amendments	
**Support**			
Sources	#5a	Indicate sources of financial or other support for the review	7
Sponsor	#5b	Provide name for the review funder and / or sponsor	7
Role of sponsor or funder	#5c	Describe roles of funder(s), sponsor(s), and / or institution(s), if any, in developing the protocol	7
**Introduction**			
Rationale	#6	Describe the rationale for the review in the context of what is already known	3
Objectives	#7	Provide an explicit statement of the question(s) the review will address with reference to participants, interventions, comparators, and outcomes (PICO)	3
**Methods**			
Eligibility criteria	#8	Specify the study characteristics (such as PICO, study design, setting, time frame) and report characteristics (such as years considered, language, publication status) to be used as criteria for eligibility for the review	4,5
Information sources	#9	Describe all intended information sources (such as electronic databases, contact with study authors, trial registers or other grey literature sources) with planned dates of coverage	4
Search strategy	#10	Present draft of search strategy to be used for at least one	4

For peer review only

		electronic database, including planned limits, such that it could be repeated	
Study records - data management	#11a	Describe the mechanism(s) that will be used to manage records and data throughout the review	5
Study records - selection process	#11b	State the process that will be used for selecting studies (such as two independent reviewers) through each phase of the review (that is, screening, eligibility and inclusion in meta-analysis)	4
Study records - data collection process	#11c	Describe planned method of extracting data from reports (such as piloting forms, done independently, in duplicate), any processes for obtaining and confirming data from investigators	5
Data items	#12	List and define all variables for which data will be sought (such as PICO items, funding sources), any pre-planned data assumptions and simplifications	5
Outcomes and prioritization	#13	List and define all outcomes for which data will be sought, including prioritization of main and additional outcomes, with rationale	5
Risk of bias in individual studies	#14	Describe anticipated methods for assessing risk of bias of individual studies, including whether this will be done at the outcome or study level, or both; state how this information will be used in data synthesis	5,6
Data synthesis	#15a	Describe criteria under which study data will be quantitatively synthesised	5,6

For peer review only

Data synthesis	#15b	If data are appropriate for quantitative synthesis, describe planned summary measures, methods of handling data and methods of combining data from studies, including any planned exploration of consistency (such as I2, Kendall's τ)	5,6
Data synthesis	#15c	Describe any proposed additional analyses (such as sensitivity or subgroup analyses, meta-regression)	N/A
Data synthesis	#15d	If quantitative synthesis is not appropriate, describe the type of summary planned	5
Meta-bias(es)	#16	Specify any planned assessment of meta-bias(es) (such as publication bias across studies, selective reporting within studies)	5
Confidence in cumulative evidence	#17	Describe how the strength of the body of evidence will be assessed (such as GRADE)	6

The PRISMA-P checklist is distributed under the terms of the Creative Commons Attribution License CC-BY 4.0. This checklist was completed on 04. October 2020 using https://www.goodreports.org/, a tool made by the EQUATOR Network in collaboration with Penelope.ai

For peer review only

BMJ Open

Personal protective equipment for reducing the risk of COVID-19 infection among healthcare workers involved in emergency trauma surgery during the pandemic: a systematic review protocol

Journal:	*BMJ Open*
Manuscript ID	bmjopen-2020-045598.R1
Article Type:	Protocol
Date Submitted by the Author:	19-Jan-2021

Complete List of Authors:	Griswold, Dylan; Cambridge University, Clinical Neurosciences<br>Gempeler, Andres; Fundación Valle del Lili, Clinical Research<br>Kolias, Angelos; Cambridge University, Neurosciences and Neurosurgery<br>Hutchinson, Peter; Cambridge University, Neurosciences and Neurosurgery<br>Rubiano, Andres M; Universidad El Bosque, Neurosciences Institute

**Primary Subject Heading**:	Research methods
Secondary Subject Heading:	Evidence based practice, Emergency medicine, Surgery, Public health, Health policy
Keywords:	COVID-19, NEUROSURGERY, ORTHOPAEDIC & TRAUMA SURGERY, Trauma management < ORTHOPAEDIC & TRAUMA SURGERY, SURGERY

SCHOLARONE™
Manuscripts

I, the Submitting Author has the right to grant and does grant on behalf of all authors of the Work (as defined in the below author licence), an exclusive licence and/or a non-exclusive licence for contributions from authors who are: i) UK Crown employees; ii) where BMJ has agreed a CC-BY licence shall apply, and/or iii) in accordance with the terms applicable for US Federal Government officers or employees acting as part of their official duties; on a worldwide, perpetual, irrevocable, royalty-free basis to BMJ Publishing Group Ltd ("BMJ") its licensees and where the relevant Journal is co-owned by BMJ to the co-owners of the Journal, to publish the Work in this journal and any other BMJ products and to exploit all rights, as set out in our licence.

The Submitting Author accepts and understands that any supply made under these terms is made by BMJ to the Submitting Author unless you are acting as an employee on behalf of your employer or a postgraduate student of an affiliated institution which is paying any applicable article publishing charge ("APC") for Open Access articles. Where the Submitting Author wishes to make the Work available on an Open Access basis (and intends to pay the relevant APC), the terms of reuse of such Open Access shall be governed by a Creative Commons licence – details of these licences and which Creative Commons licence will apply to this Work are set out in our licence referred to above.

Other than as permitted in any relevant BMJ Author's Self Archiving Policies, I confirm this Work has not been accepted for publication elsewhere, is not being considered for publication elsewhere and does not duplicate material already published. I confirm all authors consent to publication of this Work and authorise the granting of this licence.

For peer review only

1

Personal protective equipment for reducing the risk of COVID-19 infection among healthcare workers involved in emergency trauma surgery during the pandemic: an umbrella review protocol

Dylan P Griswold, BA[1,2] dg550@cam.ac.uk **Andres Gempeler, MD[3]** andres.gempeler@fvl.org.co **Angelos Kolias, MD, PhD[1,2]** ak721@cam.ac.uk **Peter J. Hutchinson, MBBS, PhD, FRCS[1,2]** pjah2@cam.ac.uk **Andres M. Rubiano, MD, PhD[1,4,5]**
1 NIHR Global Health Research Group on Neurotrauma, University of Cambridge, Cambridge, UK
2 Division of Neurosurgery, Department of Clinical Neurosciences, Addenbrooke's Hospital & University of Cambridge, Cambridge, UK 3 Clinical Research Center, Fundación Valle del Lili, Cali, Colombia4 Neuroscience Institute, INUB-MEDITECH Research Group, El Bosque University, Bogotá, Colombia 5 Neurological Surgery Service, Vallesalud Clinic, Cali, Colombia.

Keywords: systematic review; broad-evidence review; COVID-19; global health; trauma surgery; evidence-based practice; PPE; N95 masks.

Word count: 1924

Correspondence:
Andres M. Rubiano
Neuroscience Institute, INUB-MEDITECH Research Group
El Bosque University
Bogotá, Colombia
rubianoam@gmail.com

For peer review only

2

ABSTRACT

Introduction: Many healthcare facilities in low-and middle-income countries are inadequately resourced and may lack optimal organization and governance, especially concerning surgical health systems. COVID-19 has the potential to decimate these already strained surgical healthcare services unless health systems take stringent measures to protect healthcare workers from viral exposure and ensure the continuity of specialized care for the patients. The objective of this broad evidence synthesis is to identify and summarize the available literature regarding the efficacy of different personal protective equipment (PPE) for reducing the risk of COVID-19 infection in health personnel caring for patients undergoing trauma surgery in low-resource environments (LREs).

Methods We will conduct several searches in the L·OVE (Living OVerview of Evidence) platform for COVID-19, a system that performs automated regular searches in PubMed, Embase, Cochrane Central Register of Controlled Trials (CENTRAL), and over thirty other sources. The search results will be presented according to the Preferred Reporting Items for Systematic Reviews and Meta-Analyses (PRISMA) flow diagram. This review will preferentially consider systematic reviews of experimental and quasi-experimental studies, as well as individual studies of such designs, evaluating the effect of different PPE on the risk of COVID-19 infection in healthcare workers (HCWs) involved in emergency trauma surgery. Critical appraisal of the eligible studies for methodological quality will be conducted. Data will be extracted using the standardized data extraction tool in Covidence. Studies will, when possible, be pooled in a statistical meta-analysis using JBI SUMARI. The Grading of Recommendations, Assessment, Development, and Evaluation (GRADE) approach for grading the certainty of evidence will be followed, and a Summary of Findings (SoF) will be created.

Ethics and dissemination Ethical approval is not required for this review. The plan for dissemination is to publish review findings in a peer-reviewed journal and present findings at high-level conferences that engage the most pertinent stakeholders.

PROSPERO registration number CRD42020198267

For peer review only

3

ARTICLE SUMMARY

Strengths and Limitations of this study

- To the best of our knowledge this protocol provides a detailed description of the first systematic review on the effects of PPE for protection of emergency trauma surgery staff against COVID-19 infection
- The protocol adheres to the Preferred Reporting Items for Systematic Reviews and Meta-Analyses Protocols guidelines for reporting a systematic review protocol
- The protocol is being conducted by a multidisciplinary team with extensive experience of conducting high-quality systematic reviews
- Given the rate at which new COVID-19 related studies are being published, there is the possibility that new studies will have been published at the time of review publication that were not available at the time of writing the review

For peer review only

4

-

INTRODUCTION

Many healthcare facilities in low-and middle-income countries are inadequately resourced. COVID-19 has the potential to decimate these already strained surgical healthcare services unless health systems take stringent measures to protect healthcare workers (HCWs) from viral exposure. A recent study showed that 15.6% of confirmed COVID-19 patients are symptomatic and that nearly half of patients with no symptoms at detection time will develop symptoms later.[1] Furthermore, the preoperative evaluation of emergency trauma patients is limited. These factors impede and confound diagnostic triage. Improper infection prevention may create a 'super-spreader' event in a high-volume healthcare facility or reduce available personnel. Consequently, the infection control strategy of trauma surgery staff is a top priority.

To take care of patients, providers must first take care of themselves. Personal protective equipment (PPE) is paramount to protect health care workers from contracting the virus and becoming disease carriers. Basic recommended PPE for trauma surgery staff of high-income country facilities include: 1) a surgical mask or better for all personnel interacting with patients and in the OR (including cleaning staff); 2) N95 or better mask for all staff in close contact with the patients (<6 feet away); 3) powered air-purifying respirator (PAPR) for aerosolising and high-risk procedures (ear, nose, throat, thoracic, and transsphenoidal neurosurgery operations); 4) universal testing of patients pre-operatively to enable appropriate PPE use, and 5) changing scrubs after every procedure.[2] These recommendations are suitable for high-resource settings but are less feasible in low-resource settings. A rapid-turnaround survey of 40 healthcare organisations across 15 low- and middle-income countries (LMICs) revealed that 70% lack PPE and COVID-19 testing kits, and only 65% of the respondents showed confidence in hospital staff's knowledge about precautions to be taken to prevent COVID-19 infection among hospital personnel.[3]

Some resource-adjusted recommendations include the use of cloth masks and bandanas. While innovative, their moisture retention, reusability, and filtration are considered very inferior to N95, and other masks.[4] What is most needed is evidence guided recommendations for PPE use and COVID-19 screening in LMICs surgical systems where resources are either limited or unavailable. HCWs have been instructed to consider refraining from caring for patients in the absence of adequate PPE availability.

A preliminary search of PROSPERO, MEDLINE, the Cochrane Database of Systematic Reviews, and the JBI Database of Systematic Reviews and Implementation Reports was conducted, and no current or underway systematic reviews on the topic were identified.

The primary objective of the review is to summarise the effects of different personal protective equipment in reducing the risk of COVID-19 infection of health personnel caring for patients undergoing trauma surgery. There is a need for high-quality evidence in this area and a well-constructed systematic review can help provide a higher level of evidence.[5] Thus, the purpose of the review is to inform recommendations for the rational use of PPE in emergency surgery staff, particularly in low resources environments where PPE shortages and high costs are expected to hamper the safety of HCWs and affect the care of trauma patients. A preliminary search of PROSPERO, MEDLINE, the Cochrane Database of Systematic Reviews, and the JBI Database of Systematic Reviews and Implementation Reports was conducted, and no current or underway systematic reviews on the topic were identified.

METHODS

Protocol registration

A protocol of this review following the PRISMA statement was registered in the International Prospective Registry of Systematic Reviews (PROSPERO; CRD42020198267). Any changes to the protocol will be amended in PROSPERO and reported in the final review. This review was conducted following the JBI

For peer review only

methodology for systematic reviews.[6] The protocol adheres to the Preferred Reporting Items for Systematic Reviews and Meta-Analyses Protocols (PRISMA-P) 2015.[7]

Patient and public involvement

Patients and the public were not involved in the design of this systemic review protocol.

Study design

A systematic review of peer review and grey literature following the Preferred Reporting Items for Systematic Reviews and Meta-Analyses (PRISMA) approach by Moher *et al*[8] is planned for this review. Figure 1 summarises the planned stages of the review as described in this protocol.

Data source and search strategy

We will conduct several searches in the L·OVE (Living OVerview of Evidence) platform for COVID-19, a system that performs automated regular searches in PubMed, Embase, Cochrane Central Register of Controlled Trials (CENTRAL), and over thirty other sources. When compared to manual searches, this platform consistently identifies all the available studies associated with the terms of interest. It allows for a fast (automated) search that is easy to update - a crucial element given the urgent need to answer the research question rapidly and thoroughly. We will search for systematic reviews and randomized trials evaluating the effect of different personal protective equipment (PPE) on the risk of COVID-19 infection in personnel involved in emergency trauma surgery during the pandemic. Other in-hospital clinical settings will be considered for inclusion and synthesis if evidence for trauma surgery setting is not available. Different clinical settings will be treated as subgroups from which extrapolation will be possible when considered adequate. Non-randomized studies will be considered if systematic reviews and RCTs are not available or are scarce and of low quality. We will include preprint studies identified in our searches, but no ongoing studies will be considered. Ongoing studies will be counted as excluded studies in the corresponding tables and PRISMA diagram. An example search for studies involving healthcare workers and N95 masks is provided in Appendix I.

Selection of studies

Following the search, all identified citations will be collated and uploaded into EndNoteX9 (Clarivate Analytics, PA, USA). The citations will then be imported into JBI SUMARI for the review process. Two independent reviewers will examine titles and abstracts for eligibility. The full text of selected studies will be retrieved and assessed. Full-text studies that do not meet the inclusion criteria will be excluded, and a list of such excluded studies will be provided. Disagreements between the reviewers during title and abstract screening or full-text screening, will be resolved by consensus, or with a third reviewer. The results of the search will be reported in full in the final report and presented in a Preferred Reporting Items for Systematic Reviews and Meta-analyses (PRISMA) flow diagram.[8]

Eligibility criteria

Inclusion criteria

Participants

The review will preferentially include studies involving healthcare workers in emergency trauma surgery during the COVID-19 pandemic. Given the likelihood that reports on this specific population are scarce or even non-existent, if not available or insufficient we will consider studies of healthcare workers in any procedural and in-hospital setting such as ER and critical care management in COVID-19. Studies

For peer review only

summarizing the available evidence for other viral respiratory illnesses will be considered if COVID-19 evidence is not available and the setting reported is trauma surgery.

Intervention(s)

Different types of PPE used for treating patients requiring emergency trauma surgery.

Comparator(s)

Comparators of interest are no PPE use and different types of PPE.

Outcomes

This review will consider studies that include the following outcomes: Risk of contagion to health personnel involved in the care of the described population during the COVID-19 pandemic; differences in surgical field vision; expressed as incidence differences, risk ratios or odds ratios. All outcomes will be summarized narratively

Types of studies

This review will consider systematic reviews of experimental and observational studies, and experimental or observational studies if not included in systematic reviews that fulfilled population and intervention criteria. We will also include reports on implementation strategies that could inform recommendations for variable resource settings. Only studies published in English or Spanish will be included. We will include preprint studies identified in our search, but no ongoing studies will be considered.

The PICOS inclusion criteria is summarized in Table 1.

Quality assessment of included studies

Eligible studies will be critically appraised by one reviewer and verified with the other. We will use the AMSTAR tool to assess the risk of bias in systematic reviews, the Cochrane risk of bias tool (RoB2) for RCTs, and the ROBINS-I tool for non-randomized studies.[9–11] Risk of Bias will be assessed only for the primary outcome: infection of healthcare workers. The results of the risk of bias assessment will be reported narratively and inform grading of evidence summarized in SoF tables. Disagreements will be solved by consensus or by a third reviewer.

Data extraction

Data will be extracted from the included studies by a reviewer and verified by a second reviewer using a data extraction tool from JBI SUMARI.[6] The data extracted will include specific details about the populations, study methods, interventions, and outcomes of significance to the review question and specific objectives. Disagreements will be solved by consensus.

Data synthesis

Studies will be summarized narratively. Effect sizes from systematic reviews and from individual studies not included in them, will be expressed as odds ratios (for dichotomous data) with their 95% confidence intervals. Decision rules regarding data extraction for situations where data are:

For peer review only

· reported at multiple time points: include all and note time of measurement for each.

· multiple "doses": N/A – (all types of PPE will be recorded)

· multiple exposures are compared (e.g., ever exposed, frequency of exposure): as mentioned before, exposure will relate to the clinical setting of healthcare workers studied and will be classified accordingly.

For summarizing results from other settings different to trauma surgery, effect of PPE will be summarized by subgroups according to different clinical settings.

Assessing certainty in the findings

The Grading of Recommendations, Assessment, Development, and Evaluation (GRADE) approach for grading the certainty of evidence will be followed , and a Summary of Findings (SoF) will be created using online software GRADEPro GDT 2020 (McMaster University, ON, Canada).[12,13] The SoF will present the following information where appropriate: absolute risks for the treatment and control, estimates of relative risk, and quality of the evidence based on the risk of bias, directness, heterogeneity, precision, and risk of publication bias. The outcomes reported in the SoF will be risk of COVID-19 infection.

Ethics and dissemination

No ethical approval will be required, as this review is based on already published data and does not involve interaction with human subjects. The plan for dissemination, however, is to publish the findings of the review in a peer-reviewed journal and present findings at high- level international conferences that engage the most pertinent stakeholders. The proposed systematic review will provide a detailed summary of available evidence on the effects of different personal protective equipment in reducing the risk of COVID-19 infection of health personnel caring for patients undergoing trauma surgery. The purpose of the review is to inform recommendations for the rational use of PPE in emergency surgery staff, particularly in low resources environments where PPE shortages and high costs are expected to hamper the safety of HCWs and affect the care of trauma patients.

DISCUSSION

This protocol has been rigorously developed and designed specifically to assess the effects of different personal protective equipment in reducing the risk of COVID-19 infection of health personnel caring for patients undergoing trauma surgery. Given the limited recent evidence associated with the primary objective, findings from the review will be critical for researchers, policymakers, government and non-governmental organisations for planning and developing guideline recommendations for PPE use in emergency trauma surgery settings, especially in low-and middle-income countries. If protocol modifications are required, the authors will include the detailed description of any changes along with a justification during the publication of the review.

Clearly, in the era of the COVID-19, where protecting healthcare workers from infection is essential, up-to-date information on the effects of PPE for protection against COVID-19 infection is essential. This review will serve an important role as a repository of available evidence for the purpose of setting effective policy and clinical guideline recommendations.

For peer review only - http://bmjopen.bmj.com/site/about/guidelines.xhtml

8

Contributors AR, PJH, and AK conceived the review. DG, and AG designed the review. DG refined the review design. DG and AG were involved in the initial drafting of the manuscript. All authors were involved in subsequent draft manuscript reviews and updates and approved the final version of this protocol.

Funding This research was commissioned by the National Institute for Health Research (NIHR) Global Health Research Group on Neurotrauma (project 16/137/105) using UK aid from the UK Government. The views expressed in this publication are those of the author(s) and not necessarily those of the NIHR or the Department of Health and Social Care. Dr Hutchinson is the Chief Investigator of the RESCUEicp and RESCUE- ASDH randomised trials. Mr Griswold was supported by the Gates Cambridge Trust. Prof Hutchinson was supported by a Research Professorship from the NIHR, the NIHR Cambridge Biomedical Research Centre, a European Union Seventh Framework Programme grant (CENTER-TBI; grant no. 602,150), and the Royal College of Surgeons of England. Dr Kolias was supported by a Clinical Lectureship from the School of Clinical Medicine, University of Cambridge, and the Royal College of Surgeons of England. Dr Gempeler was supported by a grant from the Clinical Research Center, Fundación Valle del Lili.

Competing interests None declared.
Patient consent for publication Not required.

Data statement: This review is based on previously published data. Any relevant data will be published with the review as either an appendix or as an online supplement.

Acknowledgements None to report
Open access This is an open access article distributed in accordance with the Creative Commons Attribution Non Commercial (CC BY-NC 4.0) license, which permits others to distribute, remix, adapt, build upon this work non-commercially, and license their derivative works on different terms, provided the original work is properly cited, appropriate credit is given, any changes made indicated, and the use is non-commercial. See: http://creativecommons.org/licenses/by-nc/4.0/.

License to BMJ Group for Publication: I *Andres M. Rubiano* The Corresponding Author of this article contained within the original manuscript which includes any diagrams & photographs within and any related or stand-alone film submitted (the Contribution") has the right to grant on behalf of all authors and does grant on behalf of all authors, a licence to the BMJ Publishing Group Ltd and its licencees, to permit this Contribution (if accepted) to be published in the BMJ and any other BMJ Group products and to exploit all subsidiary rights, as set out in our licence set out at: http://www.bmj.com/about-bmj/resources-authors/forms-policies-and-checklists/copyright-open-access-and-permission-reuse.

For peer review only

9

REFERENCES

1 Zhu J, Ji P, Pang J, et al. Clinical characteristics of 3062 COVID-19 patients: A meta-analysis. J Med Virol 2020;92:1902–14. doi:10.1002/jmv.25884

2 Stewart CL, Thornblade LW, Diamond DJ, et al. Personal Protective Equipment and COVID-19: A Review for Surgeons. Ann Surg 2020;272:e132–8. doi:10.1097/sla.0000000000003991

3 Gonzalez N, Hatchcock A, Jansen N, et al. A Rapid Turnaround Survey of COVID-19 in LMICs: The Readiness, Responses, and Challenges of Healthcare Organizations. 2020.

4 R C, T D, R J, et al. Masks for Prevention of Respiratory Virus Infections, Including SARS-CoV-2, in Health Care and Community Settings: A Living Rapid Review. Ann Intern Med Published Online First: 1 January 2020. doi:10.7326/m20-3213

5 Tian Z, Stedman M, Whyte M, et al. Personal protective equipment (PPE) and infection among healthcare workers - What is the evidence? Int J Clin Pract 2020;74(11):e13617. doi: 10.1111/ijcp.13617 [published Online First: 2020/08/01]

6 Aromataris E, Munn Z, Tufanaru C, et al. JBI Reviewer's Manual. Published Online First: 2019. doi:10.46658/jbirm-17-03

7 Group P-P, Moher D, Shamseer L, et al. Preferred reporting items for systematic review and meta-analysis protocols (PRISMA-P) 2015 statement. Syst Rev 2015;4:1. doi:10.1186/2046-4053-4-1

8 Moher D, Liberati A, Tetzlaff J, et al. Preferred reporting items for systematic reviews and meta-analyses: the PRISMA statement. Int J Surg Lond Engl 2010;8:336–41. doi:10.1016/j.ijsu.2010.02.007

9 Sterne JAC, Savović J, Page MJ, et al. RoB 2: a revised tool for assessing risk of bias in randomised trials. Bmj 2019;366:l4898. doi:10.1136/bmj.l4898

10 Shea BJ, Reeves BC, Wells G, et al. AMSTAR 2: a critical appraisal tool for systematic reviews that include randomised or non-randomised studies of healthcare interventions, or both. Bmj Clin Res Ed 2017;358:j4008. doi:10.1136/bmj.j4008

11 Sterne JA, Hernán MA, Reeves BC, et al. ROBINS-I: a tool for assessing risk of bias in non-randomised studies of interventions. Bmj Clin Res Ed 2016;355:i4919. doi:10.1136/bmj.i4919

12 Atkins D, Best D, Briss PA, et al. Grading quality of evidence and strength of recommendations. Bmj 2004;328:1490. doi:10.1136/bmj.328.7454.1490

13 Guyatt G, Oxman AD, Akl EA, et al. GRADE guidelines: 1. Introduction—GRADE evidence profiles and summary of findings tables. J Clin Epidemiol 2011;64:383–94. doi:10.1016/j.jclinepi.2010.04.026

For peer review only

1

Participants	Healthcare workers in any procedural and in-hospital setting
Intervention	PPE used by emergency trauma surgery staff
Comparator	No PPE and different types of PPE within same class i.e., surgical masks vs N95
Outcomes	Risk of contagion and surgical field vision
Study Type	Systematic reviews. Experimental or observational studies not already included in the systematic reviews

Table 1. PICOS inclusion criteria

For peer review only

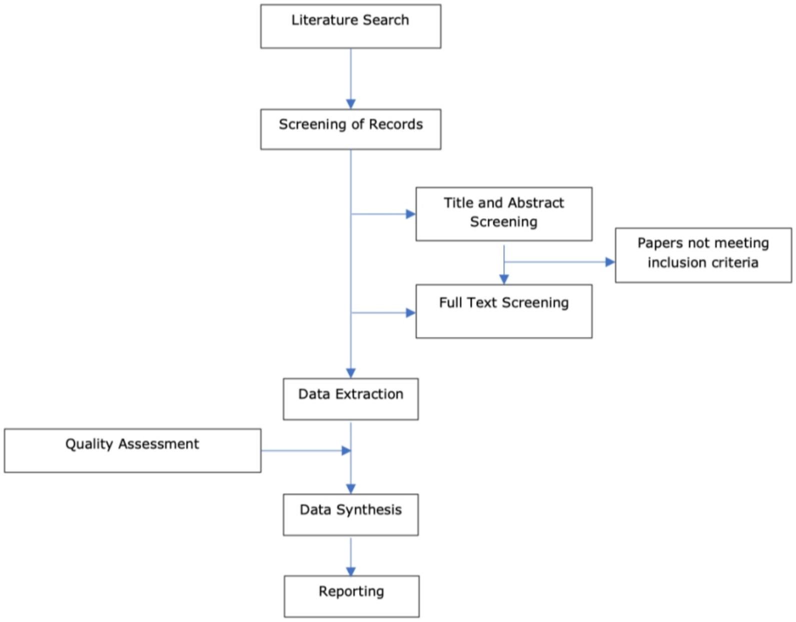

Figure 1 Summary of search strategy search process.

Figure 1. Summary of search strategy search process

1

Appendix I: The Living Overview of Evidence (L·OVE) search for COVID-19 studies involving N95 masks in healthcare workers

LoVE COVID-19 platform: https://app.iloveevidence.com/loves/5e6fdb9669c00e4ac072701d

Prevention or Treatment → Procedures → Protective measures → Personal Protective Equipment → (Options: Gloves, Shoe Covers, Masks, Eye Protection, Head Coverings, Intubation box) → Masks → Facemasks → N95 Masks

Population → Health workers → (can choose specific healthcare settings)

Results: 67 total articles included

- Broad syntheses: 3
- Systematic reviews: 10
- Primary studies: 54

The methods and report of the Special L·OVE of COVID-19 can be found using the following link: https://app.iloveevidence.com/loves/5e6fdb9669c00e4ac072701d?question_domain=5b1dcd8ae611de7ae84e8f14&population=5e9cae0f69c00e502f84a41c&intervention=5edcde1269c00e3d6aae4587&section=methods&classification=all

For peer review only

Reporting checklist for protocol of a systematic review.

		Reporting Item	Page Number
**Title**			
Identification	#1a	Identify the report as a protocol of a systematic review	1
Update	#1b	If the protocol is for an update of a previous systematic review, identify as such	NA
**Registration**			
	#2	If registered, provide the name of the registry (such as PROSPERO) and registration number	1
**Authors**			
Contact	#3a	Provide name, institutional affiliation, e-mail address of all protocol authors; provide physical mailing address of corresponding author	1
Contribution	#3b	Describe contributions of protocol authors and identify the guarantor of the review	7
**Amendments**			
	#4	If the protocol represents an amendment of a previously completed or published protocol, identify as such and list	6

For peer review only

		changes; otherwise, state plan for documenting important protocol amendments	
**Support**			
Sources	#5a	Indicate sources of financial or other support for the review	7
Sponsor	#5b	Provide name for the review funder and / or sponsor	7
Role of sponsor or funder	#5c	Describe roles of funder(s), sponsor(s), and / or institution(s), if any, in developing the protocol	7
**Introduction**			
Rationale	#6	Describe the rationale for the review in the context of what is already known	3
Objectives	#7	Provide an explicit statement of the question(s) the review will address with reference to participants, interventions, comparators, and outcomes (PICO)	3
**Methods**			
Eligibility criteria	#8	Specify the study characteristics (such as PICO, study design, setting, time frame) and report characteristics (such as years considered, language, publication status) to be used as criteria for eligibility for the review	4,5
Information sources	#9	Describe all intended information sources (such as electronic databases, contact with study authors, trial registers or other grey literature sources) with planned dates of coverage	4
Search strategy	#10	Present draft of search strategy to be used for at least one	4

For peer review only

		electronic database, including planned limits, such that it could be repeated	
Study records - data management	#11a	Describe the mechanism(s) that will be used to manage records and data throughout the review	5
Study records - selection process	#11b	State the process that will be used for selecting studies (such as two independent reviewers) through each phase of the review (that is, screening, eligibility and inclusion in meta-analysis)	4
Study records - data collection process	#11c	Describe planned method of extracting data from reports (such as piloting forms, done independently, in duplicate), any processes for obtaining and confirming data from investigators	5
Data items	#12	List and define all variables for which data will be sought (such as PICO items, funding sources), any pre-planned data assumptions and simplifications	5
Outcomes and prioritization	#13	List and define all outcomes for which data will be sought, including prioritization of main and additional outcomes, with rationale	5
Risk of bias in individual studies	#14	Describe anticipated methods for assessing risk of bias of individual studies, including whether this will be done at the outcome or study level, or both; state how this information will be used in data synthesis	5,6
Data synthesis	#15a	Describe criteria under which study data will be quantitatively synthesised	5,6

For peer review only

Data synthesis	#15b	If data are appropriate for quantitative synthesis, describe planned summary measures, methods of handling data and methods of combining data from studies, including any planned exploration of consistency (such as I2, Kendall's τ)	5,6
Data synthesis	#15c	Describe any proposed additional analyses (such as sensitivity or subgroup analyses, meta-regression)	N/A
Data synthesis	#15d	If quantitative synthesis is not appropriate, describe the type of summary planned	5
Meta-bias(es)	#16	Specify any planned assessment of meta-bias(es) (such as publication bias across studies, selective reporting within studies)	5
Confidence in cumulative evidence	#17	Describe how the strength of the body of evidence will be assessed (such as GRADE)	6

The PRISMA-P checklist is distributed under the terms of the Creative Commons Attribution License CC-BY 4.0. This checklist was completed on 04. October 2020 using https://www.goodreports.org/, a tool made by the EQUATOR Network in collaboration with Penelope.ai

For peer review only
